# The Influence of Halogenated Hypercarbon on Crystal Packing in the Series of 1-Ph-2-X-1,2-dicarba-*closo*-dodecaboranes (X = F, Cl, Br, I) †

**DOI:** 10.3390/molecules25051200

**Published:** 2020-03-06

**Authors:** Miroslav Havránek, Maksim A. Samsonov, Josef Holub, Zdeňka Růžičková, Ladislav Drož, Aleš Růžička, Jindřich Fanfrlík, Drahomír Hnyk

**Affiliations:** 1 APIGENEX s.r.o., Poděbradská 173/5, 190 00 Prague 9, Czech Republic; havranek@apigenex.com (M.H.); droz@apigenex.com (L.D.); 2 Department of General and Inorganic Chemistry, Faculty of Chemical Technology, University of Pardubice, Studentská 573, 532 10 Pardubice, Czech Republic; MaksimAndreevich.Samsonov@upce.cz; 3 Institute of Inorganic Chemistry of the Czech Academy of Sciences, 250 68 Husinec-Řež, Czech Republic; holub@iic.cas.cz; 4 Institute of Organic Chemistry and Biochemistry of the Czech Academy of Sciences, Flemingovo nám. 2, 166 10 Prague 6, Czech Republic

**Keywords:** sigma hole, halogen bond, icosahedral boron cluster

## Abstract

Although 1-Ph-2-X-*closo*-1,2-C_2_B_10_H_10_ (X = F, Cl, Br, I) derivatives had been computed to have positive values of the heat of formation, it was possible to prepare them. The corresponding solid-state structures were computationally analyzed. Electrostatic potential computations indicated the presence of highly positive σ-holes in the case of heavy halogens. Surprisingly, the halogen•••π interaction formed by the Br atom was found to be more favorable than that of I.

## 1. Introduction

Icosahedral *closo*-1,2-C_2_B_10_H_12_, known as *o*-carborane, was found to have the positive part of its relatively large value of the experimental dipole moment, 4.50 D [1], in the midpoint of the C-C vector. When one of the hypercarbon atoms is substituted with phenyl (Ph), the dipole moment is even increased to 4.93 D. The difference between these two values was interpreted as a mesomeric contribution to the overall dipole moment as a consequence of the electron transfer from the benzene ring towards the icosahedral cage. This 1-Ph-*closo*-1,2-C_2_B_10_H_11_ was structurally studied by the techniques of gas-phase electron diffraction and X-ray diffraction in the gas phase and solid state [2]. When the second hypercarbon of the cage is substituted with halogens, the overall dipole moments in the series of 1-Ph*-*2-X-*closo*-1,2-C_2_B_10_H_10_ (X = F, Cl, Br, and I, denoted here as **1**, **2**, **3**, and **4**, respectively) decrease, in the case of F and Cl by more than 1 D due to the electron-withdrawing effect of these two halogen atoms [3]. The fact that the dipole moment of Br- and I-derivatives only decreased by 0.8 and even 0.2 D, respectively, was ascribed to the close position of halogen and Ph, and also to the partially positively charged outer part of the heavy halogen atoms, known as σ-holes [4]. A σ-hole can be characterized by its magnitude, V_S,max_, defined as the value of the most positive electrostatic potential (ESP) of an electron density surface. The higher the V_S,max_ value, the more favorable the forming σ-hole interactions [5]. The halogen•••π interactions have been extensively studied in the solid state and evaluated theoretically by quantum mechanical calculations [6,7,8]. Recently, they have even been observed in solution [9]. The σ-hole interactions with aryls have been paid considerable attention in boron cluster chemistry as well, e.g., the S•••π interaction appears in 1-Ph-*closo*-1-SB_11_H_10_ [10]. 

In the series of *closo*-SB_11_H_11_ and 12-X-*closo*-SB_11_H_10_ (X = Cl, Br, I), the experimentally determined dipole moments were reported to be 3.6, 5.5, 5.5, and 5.3 D [11], respectively. Note that this series differs from the **1**−**4** series in the lack of Ph substitution and in the absence of positive σ-holes on halogen atoms (relative σ-holes occur when halogen atoms are bonded to the B vertex). Within the context of halogen•••π interactions in brominated carbaboranes [12], this bonding motif has also appeared in 1-Ph*-*2-Br-*closo*-1,2-C_2_B_10_H_10_, whose structure has been established in another laboratory [13]. In order to obtain a deeper insight into halogen•••π interactions and crystal packing within the series of **1**, **2**, **3** and **4**), we have prepared all of these halogenated 1-Ph-*o*-carboranes, crystallized them, and computationally analyzed the corresponding solid-state structures. 

## 2. Results and Discussion

### 2.1. Syntheses

Compounds **1**, **2**, **3**, and **4** were prepared by halogenating 1-phenyl-1,2-dicarba-closo-dodecaborane with *N*-fluorobenzenesulfonimide/benzene, Cl_2_/P_4_O_10_, Br_2_/toluene, and I_2_/tetrahydrofuran, respectively—as described in the literature [3]. All the compounds were identified by experimental ^11^B{^1^H} NMR spectra. The ^11^B NMR chemical shifts were compared with the theoretical shifts reported in ref. [3] (see Table 1). The largest difference of 3.4 ppm was found for B4 and B5 of **4**. The purities of **1**–**4** were checked by analytical TLC. 

### 2.2. Structural Characterization

**Figure 1:**
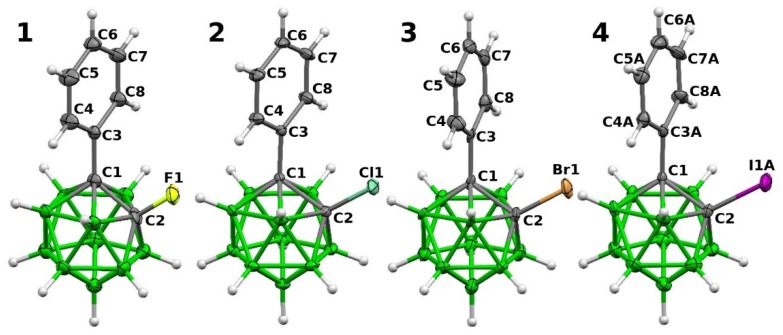
The molecular structures of complexes **1**–**4**. Thermal ellipsoids are at the 40% probability level.

**Figure 2:**
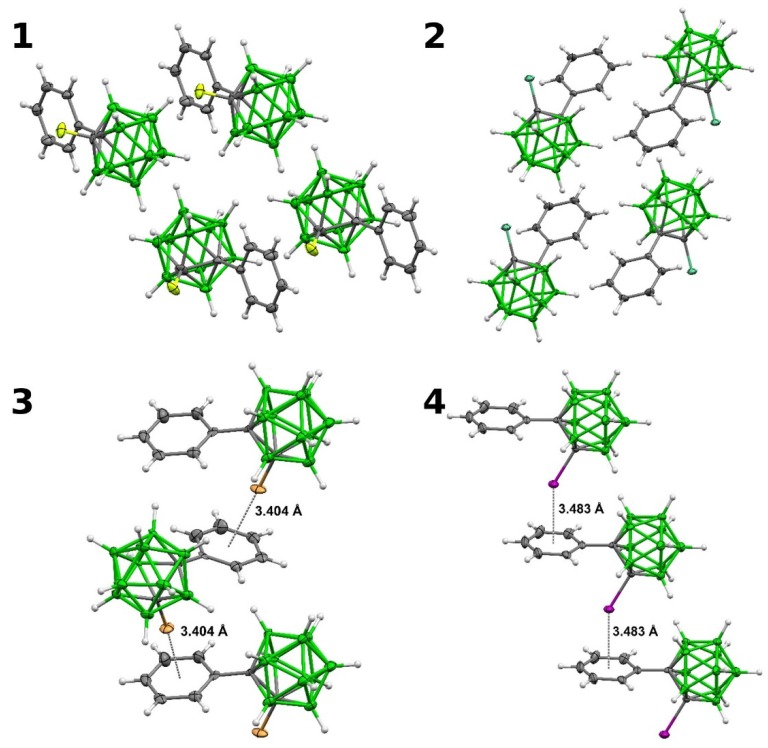
**A** fragment of crystal packing in **1**–**4**. Thermal ellipsoids are at the 40% probability level.

The crystal structure of **3** had previously been reported by Welch as well [13]. It contained a noticeable interaction between the Br atom and the Ph ring (Br•••C contacts of 3.457 and 3.488 Å, the Br•••Ph_center_ separation of 3.463 Å and the C-Br•••Ph_center_ angle of 175.63°). We have solved the single-crystal structures of **1**, **2**, **3**, and **4** (Figure 1 and Figure 2). The newly reported crystal structure of **3** confirms the formation of a characteristic interaction between the Br atom and the Ph ring (the Br1•••C7, Br1•••C8 and Br1•••Ph_centroid_ separations of 3.416, 3.436, and 3.404 Å, respectively, and the C2-Br1•••Ph_center_ angle of 175.14°). The interaction between the I atom and the Ph ring was of comparable length (the I1B•••C7B, I1B•••C8B and I1B•••Ph_center_ separations of 3.553, 3.594, and 3.483 Å) but considerably more bent (C-I•••Ph_center_ angle of 145.00°). 

The unit cell of **4** contains two independent molecules. The molecular structures of all complexes **1**–**4** are similar (Figure 1). The distances C1-C2 and C1-C3(Ph) lie within the intervals (1.620(13)–1.706(2) Å) and (1.476(16)–1.505(9) Å), respectively. The largest geometric differences are observed when the relative position of phenyl rings is analyzed. The torsion angle C2-C1-C3-C8 is 70.5(6)° and 72.0(2)° in the case of **1** and **2**, respectively, 87.3(13)° for **3,** and 84.9(11)° and 82.9(11)° for **4**. Such differences can be explained by the features of crystal packing and the presence of intermolecular interactions C-X•••π in the case of Br- and I-derivatives, not observed in F- and Cl-derivatives (Figure 2), where usual B-H•••X and C-H•••H-B connections prevail.

### 2.3. Computations

#### 2.3.1. Heat of Formation (ΔH_f_^298^)

The computed ΔH_f_^298^ values of the studied compounds and their 10-vertex analogues are summarized in Table 2. Since all the considered compounds have a high energy level (positive ΔH_f_^298^ values), their thermodynamic stability should be low as they can lose a great deal of energy by reacting to lower-energy products. The thermodynamic stability has decreased with the increasing atomic number of the halogen atom and with the reduced size of the carborane cage. Note that the positive values of the heat of formation do not necessarily mean experimental unavailability, as exemplified by e.g., *closo*-SB_9_H_9_ with the computed ΔH_f_^298^ value of 11.3 kcal mol^−1^ [14,15]. Moreover, we have computed the ΔH_f_^298^ of 26.5 kcal mol^−1^ for *closo*-1,2-C_2_B_8_H_10_, which was previously prepared as well [16].

#### 2.3.2. Electrostatic Potential (ESP)

**Figure 3:**
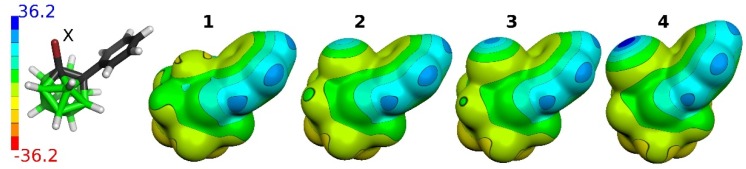
The computed electrostatic potential (ESP) molecular surfaces of the **1**, **2**, **3**, and **4** compounds. The ESP has been computed at the HF/def2TZVP level. The ESP color range is in kcal mol^−1^

As the studied compounds are neutral and the halogen atoms are bound to a C-vertex, one can expect the heavier halogen atoms with highly positive σ-holes. The molecular ESP surfaces of the studied molecules were computed in order to validate this assumption (see Figure 3). Indeed, the top of the I and Br atoms with the V_S,max_ values of 36.2 and 25.8 kcal mol^−1^, respectively, is the most positive part of the **3** and **4** molecules. In the case of **2**, the σ-hole of the Cl atom has the V_S,max_ value of 19.5 kcal mol^−1^. The H atoms of the Ph ring are thus more positive (V_S,max_ value of 23.9 kcal mol^−1^). The F atom of **1** does not have a positive σ-hole (the V_S,max_ value of −5.7 kcal mol^−1^) due to its large electronegativity. The most negative values (V_S,min_) of the molecular surfaces of the studied molecules are on BH(9) vertices, which are antipodal to CX(2) vertices. The V_S,min_ values range from −14.0 to −12.9 kcal mol^−1^ (for **4** and **1** compounds, respectively). Besides hydridic BH vertices, the σ-holes on the heavier halogen can also interact with the π electrons of Ph rings, which have a negative ESP surface as well (the V_S,min_ values range between −6.8 and −6.4 kcal mol^−1^ for **4** and **2** compounds, respectively). 

#### 2.3.3. Interactions in the Single Crystals of **1**–**4**

Interactions in the reported single-crystal structures were studied by computing two-body and many-body interaction energy (ΔE^2^ and ΔE^MB^) values between the central molecules and two layers of surroundings molecules. The first layer consisted of molecules within 5 Å of the central molecule, and the second layer was formed by molecules within 5 Å of the first layer. The obtained sums of the ΔE values are summarized in Table 3. The computed total binding became more favorable with the increasing atomic number of the halogen atom (i.e. −48.4, −51.4, −53.3, and −55.2 kcal mol^−1^ for F-, Cl-, Br-, and I-containing compounds, respectively). The total binding thus correlated more with the molecular masses of these molecules (R^2^ of 0.91) than with their experimental dipole moments (R^2^ of 0.76). 

**Figure 4:**
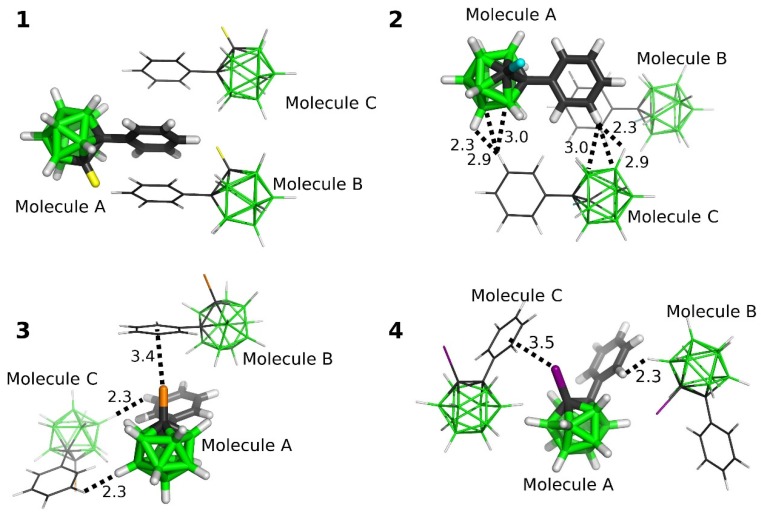
The most stable interaction motifs from the studied crystals of the **1**–**4** compounds. The positions of H atoms have been optimized at the DFT-D3/BLYP/DZVP level.

The interaction motifs with the most favorable ΔE^2^ values are shown in Figure 4 and the corresponding values in Table 4. This analysis confirmed the strength of the halogen•••π interaction of **3**—the motif with the C-Br•••Ph interaction had the ΔE^2^ of −6.91 kcal mol^−1^ at the DFT-D3 level. Considering that each molecule of **3** formed two such C-Br•••Ph interactions, it thus accounted for about 26% of the total computed binding of **3**. According to the SAPT0 decomposition, this motif was mainly stabilized by dispersion, which formed approximately 65 of the attractive terms. The second most important term was electrostatic. It formed about 28% of the attractive terms, which was the largest contribution to the electrostatic term among all the motifs studied (see Table 4). The second most favorable motif of **3** had two diH-bonds and contributed considerably less to the overall binding. With the ΔE^2^ of −5.67 kcal mol^−1^, it only formed about 11% of the total binding of **3**. 

In the case of **4**, the motif with the halogen•••π interaction had the ΔE^2^ of −5.79 kcal mol^−1^ and thus formed about 21% of the overall binding of **4**. Therefore, it was less favorable than the motif with the halogen•••π interaction of **3**. It was surprising considering the large V_S,max_ value of **4** (see part 2.3.2.). However, examples of a reverse hierarchy in strength of halogen interactions have already been reported in literature [17,18]. In our case, the lack of strength of the I•••π interaction corresponded to the bent C-I•••Ph_center_ angle. An optimal arrangement for a σ-hole hole interaction is linear, whereas the C-I•••Ph_center_ angle was about 145° in the case of **4**. Additionally, we have modeled a hypothetical dimer of **4** stabilized by a halogen•••π interaction in an optimal arrangement. The obtained motif had the I•••Ph_center_ separation of 3.6 Å, the C-I•••Ph_center_ angle of 170° and the ΔE^2^ of −8.50 kcal mol^−1^ at the DFT-D3 level, which demonstrated the capability of **4** to form a very strong iodine•••π interaction. These results indicated that crystal packing effects made the C-I•••π interaction of **4** weaker. Consequently, the most favorable crystallographic motif of **4** was the motif A•••B, which had the diH-bond with the length of 2.26 Å and the ΔE^2^ value of −5.98 kcal mol^−1^.

The most favorable motif of **2** had the highly negative ΔE^2^ of −7.10 kcal mol^−1^ (the most negative ΔE^2^ of this study). This motif formed about 14% of the total binding of **2** and did not have any close contact below the sum of van der Waals radii. The motif can be characterized by a large dispersion term, which formed about 73% of the attractive terms of the SAPT (Symmetry Adapted Perturbation Theory) decomposition. The second most favorable motif of **2** had multiple diH-bonds, the ΔE^2^ of −7.10 kcal mol^−1^ and a large dispersion term (i.e., about 75% of the attractive terms).

The two most favorable motifs of **1** had comparable ΔE^2^ of −6.93 and −6.58 kcal mol^−1^. Together, they formed about 28% of the total binding of **1**. Neither of them formed close contact below the sum of van der Waals radii, and both had a large dispersion term in the SAPT decomposition, i.e., the dispersion term ranged from 75 to 80% of the attractive terms. 

### 2.4. Cambridge Structural Database (CSD) search

**Figure 5:**
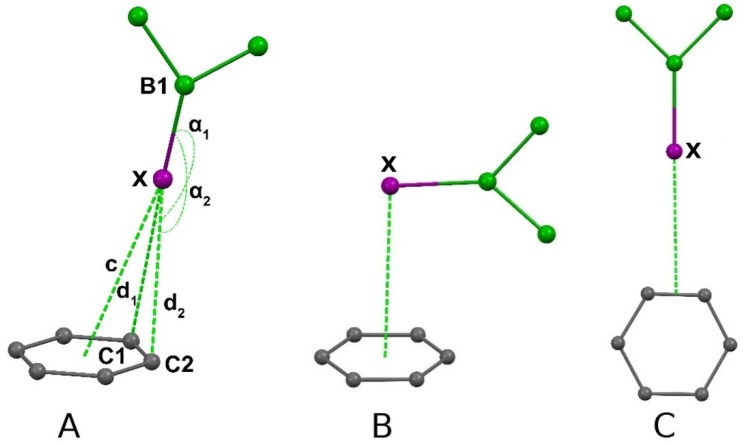
A general scheme of the fragment used in the analysis of the CCDC and some examples of the structural motifs found. The **A**, **B**, **C** are the fragment used.

We have searched the Cambridge structural database (CSD) [19] for X-ray structures containing halogenated carboranes that exhibit interactions between a halogen and a Ph ring. The analysis of CSD [19], however, did not show any analogous halogen•••Ph interactions in similar ortho-carborane derivatives. Additionally, we analyzed short B-X•••Ph-ring contacts in various halogenated boron compounds (for the definition of the criteria, see Figure 5). We fixed d_1_ and d_2_ to be less than sum of van der Waals radii of the appropriate elements [20], as well as angles B1-X•••C1,2 (90° < α1, α2 < 180°). A minor number of hits was excluded as clearly not suitable for the criteria of this type of interaction. The data are presented in Table 5; the found contacts may be potential candidates for studies of unusual B-X•••π interactions.

Specifically, the set of four fluorine-substituted compounds mostly contain the side-on intermolecularly interacting compounds with the B-Hal bond lying in the plane of the aromatic ring (Figure 5C). This could be attributed more or less to the non-classical C-H•••X hydrogen bond [21,22]. Nineteen relevant chlorine-substituted compounds exhibit mainly contacts contrived by C-H•••Cl or B-H•••H-C interactions, and only four structures of ionic compounds (halogenated carbadodecaborate anions are compensated by tritylium, silylium, and borinium cations), which are considered products of Cl•••π interactions [23,24,25]. A different situation occurs for brominated compounds, where the Br•••π interaction has been found to be dominant in most compounds in a set selected based on defined parameters, except for a couple of examples of type C and B (Figure 5) and boarder-line cases [26]. Surprisingly enough, only three relevant compounds have been found in the set of iodo compounds [27,28,29,30,31,32,33]. To conclude here, the most probable is an interaction of the desired type in brominated and iodinated compounds, where the aromatic ring is not a part of the same moiety as the halogen. The desired criteria are also accomplished for chlorinated ionic compounds, where the weak nucleophiles, such as CHB_11_X_11_^−^, are compensated for by aromatic ring-containing cations. If all the restrictions were removed, leaving only the Ph ring, halogen, and three boron atoms, then 418 hits would be obtained. One can hence assume that the probability of the formation of such a motif in crystals containing both halogenated boranes and aromatic systems is 8.9%. 

## 3. Materials and Methods 

### 3.1. X-Ray Crystallography

The X-ray data for the compounds **1**–**4** (colorless crystals obtained by slow evaporation of a hexane solution) were collected at 150(2)K with a Bruker D8-Venture diffractometer equipped with a Mo (Mo/K_α_ radiation; λ = 0.71073 Å) microfocus X-ray (IµS) source, by a Photon CMOS detector and an Oxford Cryosystems cooling device. The frames were integrated with the Bruker SAINT software package [34] using a narrow-frame algorithm. The data were corrected for absorption effects using the Multi-Scan method (SADABS) [35]. The obtained data were treated by XT-version 2014/5 [36] and SHELXL-2017/1 [37] software implemented in the APEX3 v2018.1-0 (Bruker AXS Inc., Madison, WI, USA) system. Compound **1** exhibits a disorder of B(5)-H(5) and C(2)-F(1) groups (50:50). H atoms were placed in calculated positions and refined in the “riding model”. R_int_ = Σ_|_F_o_^2^ − F_o,mean_^2^_|_/ΣF_o_^2^, S = [Σ (w(F_o_^2^ − F_c_^2^)^2^)/(N_diffrs_ − N_params_)]^½^ for all data, R(F) = Σ_||_Fo_|_ − _|_Fc_||_/Σ_|_F_o|_ for observed data, wR(F^2^) = [Σ(w(F_o_^2^ − F_c_^2^)^2^)/(Σw(F_o_^2^)^2^)]^½^ for all data. Crystallographic data for the structural analysis have been deposited with the Cambridge Crystallographic Data Centre CCDC no. 1981411–1981414. Copies of this information may be obtained free of charge from The Director, CCDC, 12 Union Road, Cambridge CB2 1EY, UK (fax: +44-1223-336033; email: deposit@ccdc.cam.ac.uk or www: http://www.ccdc.cam.ac.uk). For experimental findings see Table 6.

### 3.2. Computations

#### 3.2.1. Electrostatic Potential (ESP)

The molecular ESP surfaces were computed on the 0.001 a.u. molecular surfaces at the HF/def2-TZVP level using the Gaussian09 [38] and Molekel4.3 [39,40] programs.

#### 3.2.2. Heat of Formation (ΔH_f_^298^)

For optimization, we used the DFT/B-P86/def2QZVP level and the LBFGS algorithm with strict optimization criteria (i.e. ΔE < 0.0006 kcal mol^−1^, the maximal gradient <0.12 kcal mol^−1^ Å^−1^ and the RMS of gradient <0.06 kcal mol^−1^ Å^−1^. Harmonic vibrational calculations for the ZPVE and other thermodynamic contributions were computed at the DFT/B-P86/def2QZVP level. The energy calculations of the studied molecules and the atoms they constitute were performed at the DFT/B3LYP/def2QZVP level. ΔH_f_^298^ values were computed by Cuby4 [41] program package, which called Turbomole 7.0 [42] for a harmonic vibrational and energy calculations.

#### 3.2.3. Interaction Energy

The interactions of the crystal structures were studied by using a cluster model. Hydrogen atoms of the central molecule and the surrounding molecules of the first layer were optimized by the DFT-D3/BLYP/DZVP method [43]. The resolution-of-identity (RI) approximation to the DFT method was used. Hydrogen atoms of the surrounding molecules of the second layer were optimized by the semiempirical quantum mechanical PM6-D3H4X method [44]. Heavy atoms were kept in crystallographic positions. Interaction energies were computed at the DFT-D3/TPSS/TZVPP level. Two-body interaction energy (ΔE^2^) was computed as the energy difference between the energy of the dimer and the sum of monomer energies. For the first layer, the interaction energy between the central molecule and the whole first layer (ΔE(AQ)) was computed as well. The many-body interaction energy (ΔE^MB^) was computed as the difference between ΔE(AQ) and the sum of ΔE^2^ values. ΔE values of selected motifs were decomposed using symmetry-adapted perturbation-theory (SAPT) methodology. The simplest truncation of SAPT (SAPT0) decomposition [45] was performed with the recommended jun-cc-pVDZ basis set [46]. Turbomole (7.0) [42], MOPAC2016 [47], PSI4 [48], and Cuby4 [41] programs were used.

## 4. Conclusions

A series of **1**−**4** derivatives was prepared, crystallized, and computationally analyzed. Even though their heat of formation had been computed to be positive, it was possible to prepare them. The obtained solid-state structures were computationally analyzed and the presence of σ-holes in the case of heavy halogens was computationally established. Interestingly, the halogen•••π interaction coming from the Br atom was found to be more favorable than that of I.

## Figures and Tables

**Table 1:** ^11^B chemical shifts (in ppm) for **1**–**4** with respect to BF_3_.OEt_2_. ^11^B NMR spectra were recorded on a Varian Unity—500 instrument in CDCl_3_ solution. Computed shifts taken from ref. [3] are shown in parentheses. The calculations were performed at the GIAO-B3LYP/II//MP2/6-31G* level (DZP + ECP were used for **3**–**4**).

Compound	B9	B12	B4, B5	B7, B11	B3, B6	B8, B10
**1**	−6.6 (−7.1)	−11.2 (−11.3)	−12.6 (−14.0)	−13.6 (−15.6)	−14.6 (−16.1)	−14.6 (−16.4)
**2**	−4.6 (−5.1)	−6.4 (−6.8)	−10.2 (−11.6)	−10.2 (−11.6)	−10.7 (−12.3)	−11.8 (−13.5)
**3**	−4.2 (−4.8)	−5.3 (−6.0)	−9.2 (−11.2)	−10.8 (−12.0)	−10.8 (−12.8)	−10.8 (−13.1)
**4**	−3.2 (−4.4)	−3.7 (−4.5)	−7.8 (−11.2)	−9.4 (−11.6)	−9.4 (−12.6)	−10.2 (−12.8)

**Table 2:** The computed heats of formation (ΔH_f_^298^) in kcal mol^−1^.

Compound	ΔH_f_^298^
12-vertex series	
1-Ph-2-F-*closo*-1,2-C_2_B_10_H_10_ (**1**)	2.7
1-Ph-2-Cl-*closo*-1,2-C_2_B_10_H_10_ (**2**)	45.7
1-Ph-2-Br-*closo*-1,2-C_2_B_10_H_10_ (**3**)	57.0
1-Ph-2-I-*closo*-1,2-C_2_B_10_H_10_ (**4**)	64.9
10-vertex series	
1-Ph-2-F-*closo*-1,2-C_2_B_8_H_8_	30.9
1-Ph-2-Cl-*closo*-1,2-C_2_B_8_H_8_	73.2
1-Ph-2-Br-*closo*-1,2-C_2_B_8_H_8_	84.6
1-Ph-2-I-*closo*-1,2-C_2_B_8_H_8_	92.6

**Table 3:** Two-body and many-body interaction energies (ΔE^2^ and ΔE^MB^) computed at the DFT-D3/TPSS/TZVPP level in kcal mol^−1^.

Compound	ΣΔE^2^ (1st Layer)	ΔE^MB^ (1st Layer)	ΣΔE^2^ (2nd Layer)	Total
1-Ph-2-F-*closo*-1,2-C_2_B_10_H_10_ (**1**)	−50.74	5.04	−2.69	−48.39
1-Ph-2-Cl-*closo*-1,2-C_2_B_10_H_10_ (**2**)	−52.29	5.51	−4.60	−51.38
1-Ph-2-Br-*closo*-1,2-C_2_B_10_H_10_ (**3**)	−53.72	4.05	−3.58	−53.25
1-Ph-2-I-*closo*-1,2-C_2_B_10_H_10_ (**4**)	−56.31	3.45	−2.37	−55.23

**Table 4:** Interaction energies computed at the DFT-D3/TPSS/TZVPP level. The interaction energies have been decomposed into electrostatic (Eelec), induction (Eind), dispersion (Edisp), and exchange (Eexch) contributions using the SAPT0/jun-cc-pVDZ methodology. All energies are in kcal mol^−1^. The relative values in parentheses show the contribution to the sum of all the attractive energy terms of SAPT.

Motif	DFT-D3	SAPT0				
		Total	E_elec_	E_ind_	E_disp_	E_exch_
1-Ph-2-F-*closo*-1,2-C_2_B_10_H_10_ (**1**)						
A•••B	−6.93	−7.20	−1.92 (15.8%)	−0.57 (4.7%)	−9.65 (79.5%)	4.94
A•••C	−6.58	−7.15	−2.56 (19.5%)	−0.68 (5.2%)	−9.87 (75.3%)	5.96
1-Ph-2-Cl-*closo*-1,2-C_2_B_10_H_10_ (**2**)						
A•••B	−7.10	−7.82	−3.44 (21.5%)	−0.91 (5.7%)	−11.63 (72.8%)	8.15
A•••C	−5.43	−4.64	−1.68 (15.9%)	−1.03 (9.8 %)	−7.87 (74.4%)	5.95
1-Ph-2-Br-*closo*-1,2-C_2_B_10_H_10_ (**3**)						
A•••B^1^	−6.91	−7.10	−4.80 (27.6%)	−1.27 (7.3%)	−11.30 (65.1%)	10.23
A•••C^2^	−5.67	−5.27	−2.19 (22.6%)	−0.73 (7.5%)	−6.75 (69.8%)	4.40
1-Ph-2-I-*closo*-1,2-C_2_B_10_H_10_ (**4**)						
A•••B	−5.98	−	−	−	−	−
A•••C	−5.79	−	−	−	−	−

^1^ The analogous motif of the crystal structure by A. Welch et al. [13] was computed to have the interaction energy of −6.73 kcal mol^−1^ at the MP2/CBS level [12]. ^2^ The analogous motif of the crystal structure by Welch [13] was computed to have the interaction energy of −5.09 kcal mol^−1^ at the MP2/CBS level [12].

**Table 5:** The data obtained in the analysis of the fragment in the CCDC.

X	Restraints, Å	Number of Structures	Angles (B-Hal-C) α_1_, α_2_,°	d_1_, d_2_, Å	c, Å
F	2.8 < (d_1_, d_2_) < 3.1	4	99.08–168.80	2.871–3.090	3.235–4.013
Cl	3.1 < (d_1_, d_2_) < 3.5	19	96.77–169.76	3.156–3.497	3.172–4.287
Br	3.1 < (d_1_, d_2_) < 3.6	10	104.44–171.01	3.329–3.586	3.567–4.476
I	3.1 < (d_1_, d_2_) < 3.8	4	140.21–176.25	3.437–3.795	3.416–4.078

**Table 6:** The refinement information and crystallographic data for **1**–**4**.

Compound	1	2	3	4
Chemical formula	C_8_H_15_B_10_F	C_8_H_15_B_10_Cl	C_8_H_15_B_10_Br	C_8_H_15_B_10_I
Formula weight	238.30	254.75	299.21	346.20
Temperature/K	150(2)	150(2)	150(2)	150(2)
Crystal system	Monoclinic	Monoclinic	Orthorhombic	Monoclinic
Space group	*P*2_1_/m	*P*2_1_/n	*Pbca*	*P*2_1_
a/Å	8.6564(9)	7.2920(4)	10.257(3)	12.0950(8)
b/Å	7.5229(7)	23.9912(14)	11.448(3)	7.2033(5)
c/Å	10.5576(11)	7.7979(5)	24.132(7)	16.9983(14)
α/°	90	90	90	90
β/°	106.168(3)	93.397(2)	90	90.453(3)
γ/°	90	90	90	90
Volume/Å	660.33(12)	1361.80(14)	2833.8(14)	1480.91(19)
Z	2	4	8	4
ρcalc g/cm^3^	1.199	1.243	1.403	1.553
μ/mm^−1^	0.066	0.248	2.870	2.133
F(000)	244	520	1184	664
Crystal size/mm^3^	0.989 × 0.504 × 0.386	0.414 × 0.225 × 0.148	0.342 × 0.192 × 0.150	0.753 × 0.416 × 0.343
Radiation type	MoKα (λ = 0.71073 Å)	MoKα (λ = 0.71073 Å)	MoKα (λ = 0.71073 Å)	MoKα (λ = 0.71073 Å)
2θ range for data collection/°	2.450 to 27.996	2.751 to 26.415	2.606 to 24.999	2.396 to 28.276
Index ranges	−11 < = h < =11, −9 < = k < = 9, −13 < = l < = 13	−9 < = h < = 9, −29 < = k < = 30, −9 < = l < = 9	−12 < = h < = 12, −13 < = k < = 12, −28 < = l < = 28	−14 < = h < = 16, −9 < = k < = 9, −22 < = l < = 22
Reflections collected	14986	33108	14758	21597
Independent reflections	1704 [R(int) = 0.0745]	2794 [R(int) = 0.0694]	2439 [R(int) = 0.1169]	6917 [R(int) = 0.0499]
Data/restraints/parameters	1704/12/115	2794/0/172	2439/264/172	6917/1/344
Goodness-of-fit on F2	1.049	1.067	1.176	1.053
Final R indexes [I > 2σ(I)]	*R*_1_ = 0.0620, *wR*_2_ = 0.1615	*R*_1_ = 0.0479, *wR*_2_ = 0.1096	*R*_1_ = 0.1237, *wR*_2_ = 0.2686	*R*_1_ = 0.0370, *wR*_2_ = 0.0718
Largest diff. peak/hole/e Å-3	0.673 and −0.393	0.282 and −0.307	1.683 and −1.521	1.635 and −1.431
